# Progress of Surgery

**Published:** 1875-06

**Authors:** Jno. E. Owens

**Affiliations:** Lecturer on Surgery, Rush Medical College, Chicago


### Progress in Medical Sciences.

ARTICLE I.

### PROGRESS OF SURGERY.

BY JNO. E. OWENS, M.D.,

LECTURER ON SURGERY, RUSH MEDICAL COLLEGE, CHICAGO.

1. Alleged Rotation of the Ulna. By THOMAS DWIGHT, Jr., M.D. (*The Boston Medical and Surgical Journal*, March 11, 1875.)

2. Salicylic Acid. (*The Boston Medical and Surgical Journal*, March 11, 1875.)

3. Operative Means for the Relief of Patients Suffering with Advanced Prostatic Disease. By Sir HENRY THOMPSON. (*London Lancet*, March, 1875.)

4. Cotton-Wool Dressing for Wounds. (*The Boston Medical and Surgical Journal*, March 18, 1875.)

1. Dr. Dwight denies the alleged rotation of the ulna, "as held by Dr. Lecompte."* The demonstration of the latter consists in inclosing the wrist in a metal ring large enough to allow it to turn, and in noticing that as the hand is rotated the end of the ulna undoubtedly changes its place. It is hard to believe, says Dr. Dwight, that the ulna does not move; but the following experiment, which he has tried several times, shows conclusively that the appearance is deceptive: Rotate the arm of a cadaver, and observe the same apparent movement of the bone that is seen in life; then make a small incision down to the bone on the styloid process of the ulna, and drive a large pin firmly in. On fixing the humerus and repeating the rotation, it will be seen that the pin does not move.

2. Upon the authority of Prof. Kolbe, salicylic acid has been employed, in the lying-in hospital of Leipsic, to the exclusion of carbolic acid, since July last, for dis-

* Archives Generales de Medecine, August, 1874.

infection of the hands, in vaginal douching, etc., in solution in water of one part in three hundred to one part in nine hundred, or as a powder mixed with starch in proportion of one part in five. This use of salicylic acid has been attended with such successful results, that it is recommended in the strongest terms for use in obstetric practice, by the authorities of the hospital. Prof. Kolbe suggests that physicians, and especially hospital physicians, should study the action of salicylic as a medicine, whether and in what quantity of larger or lesser doses it will influence scarlet fever, diphtheria, eruptions, syphilis, etc.; and whether it may be used against *pyæmia* and the *bites* of *dogs*. To prove the innocuousness of salicylic acid, Kolbe took, for several consecutive days, seven and a half grains daily in water, (one part to one thousand) without the slightest observable unpleasant effect. After an interval of eight days he took for five consecutive days fifteen and a half grains daily, and then for two days twenty-three grains in alcohol each day. The digestion was perfectly normal; no trace of salicylic acid could be found in the urine or fæces. (Perchloride, the test, gives an intense violet color). At no time was there the slightest discomfort. Prof. Kolbe and eight of his students repeated the experiment. Each took on the first day one gramme (15½ grs.), and on the second day one and a quarter grammes, of salicylic acid. Not one of them was able to observe the slightest derangement of any organs.

C. Neubauer (a pupil of Prof. Kolbe) has experimented with salicylic acid to determine the quantity necessary to arrest fermentation in solutions of sugar and in new wine. He found that one gramme of salicylic acid is adequate to make 0.98 gramme of pure yeast (weighed dry) in ten litres (about ten quarts) of new wine incapable of fermentation.

We anticipate gratifying results from the employment of salicylic acid in surgery.

3. Sir Henry Thompson adds another method of relief to patients suffering from "advanced prostatic disease." By this term, the author refers to the last stage of complete or permanent obstruction existing at or about the neck of the bladder—obstruction to the outflow of urine by natural efforts. When obstruction is not only complete but permanent, disease of the prostate is mostly the cause. Having already gone over the ground, Sir Henry merely reminds his audience (at University College Hospital, London,) that very many individuals between fifty-five and sixty years of age begin to be unable to expel entirely the contents of the bladder. The act of micturition is more frequently performed, and requires some effort; the stream grows feeble, and, under the influence of external cold, or of some diuretic act distending the bladder, or other cause, it often happens that complete or nearly complete retention is established. In such cases of hypertrophy, the bladder is generally not emptied at any act; and if, immediately after an attempt to do so, a catheter be passed, a notable quantity will be left behind, and is drawn off by the instrument. Under these circumstances the patient is taught to use the catheter twice, three times, or more frequently, according to his necessity, in the twenty-four hours. With increase in age the bladder often requires relief six, seven or eight times daily. In some cases, which happily are only exceptional, a more advanced stage arrives, by gradual steps, associated with a greatly diminished capacity of the bladder, so that the use of the catheter becomes necessary from sixteen to twenty-four times, or more, in the twenty-four hours. One can easily imagine the extreme misery, loss of rest, fatigue which such a condition entails. At such a stage, the augmented volume of the prostate sometimes makes the canal difficult to traverse alike for the patient and the surgeon. Should a false passage be made at this crisis, the result is almost necessarily fatal. Such a patient then exists for little else than to pass his catheter. No sooner has he obtained a half hour's relief, than he begins to feel the warning of another approaching call. His powers are tasked to the utmost, continuous sleep is out of the question, and health rapidly declines.

The method of relief referred to consists of a proceeding similar to that which we employ in the trachea when death is imminent for want of air, viz., the introduction of a tube beyond the seat of obstruction—one which is to be permanently retained there as the constant channel for urine, just as the tracheotomy tube has been for many a patient the only channel during years for air to the lungs. The proceeding proposed, the puncture of the bladder behind the pubes, differs from ordinary puncture, and more resembles the high operation for stone, being rather a compound of the two. The operation consists in passing a large sound, hollow throughout, with a strongly marked curve, and closed by a bulbous-ended stylet. The instrument is introduced by the urethra until the end can be felt just behind the symphysis pubis. It is then confided to an assistant to retain in its place. The operator now makes an incision not more than three-quarters of an inch in length, less if the patient is not stout,—enough to admit the index finger tightly, since a larger opening becomes embarrassing subsequently,—in the median line at the upper margin of the symphysis. The tissues are separated by the finger, and the linea alba being next slightly divided by the point of a bistoury, the finger is passed down closely behind the symphysis, and when the end of the sound is clearly felt, a little opening is made, in order to expose its point. The operator now taking the handle of the sound in his left hand, makes the end protrude in the wound, the bulbous stylet is withdrawn, and he passes a tube in its whole length into the hollow channel of the sound. The supra-pubic tube, (elastic gum) is about two and a half inches long, and fitted to a silver plate. The withdrawal of the sound leaves the tube in the bladder. The tube must be securely fastened with tapes and plaster, and worn a few days in bed until the parts are consolidated, after which the patient can move about with safety.

It is important that the wound be as small as possible. A large wound is more painful, and is constantly traversed by the urine.

4. G. B. S., in his "Letters from Paris," reports favorably upon the value of M. Guerin's cotton-wool dressing for wounds. He reminds us that this dressing has now been in use about five years in Paris, and may be fairly considered "to have made its proofs." Last May, Monsieur Guerin submitted to the Academie des Sciences a paper upon the influences of ferments in surgical maladies, upon which paper a committee, composed of such men as Claude Bernard, Pasteur, Sedillot and Larrey, lately presented a report. The Societe de Chirurgie, at a late meeting, decreed the Duval prize to M. Hervey, a former "interne" of Guerin for a very elaborate paper upon cotton-wool dressing in practice. The paper of the latter considered simply the theory of the dressing.

However much the difference of opinion among the best authorities as to the theory, the practical usefulness of cotton-wool as a dressing is generally accepted.

M. Guerin expressed himself to the correspondent as continuing entirely convinced of the merits of his system, and quite content to await the verdict of posterity. He insists upon the action of the cotton as a filter; the air reaches the wound, but leaves upon the way its noxious accompaniments; the pus beneath the cotton undergoes a chemical decomposition, entirely different from putrid fermentation. The pus, examined under the microscope, is found to have lost its globules, and presents the appearance of a fatty emulsion.

A quantity of fresh pus was carefully inclosed in cotton-wool, in such a manner as to avoid previous contact with the external air. At the end of forty-two days it had no putrid odor, and microscopically showed no traces of any living corpuscles; there were neither bacteria nor vibriones.

Another portion of pus, taken with less care, and preserved beneath a less thick layer of cotton, gave out at the end of three weeks the usual putrid smell, and showed great numbers of vibriones.

He has renounced the earlier practice of wetting the dressings with disinfecting solutions, as it injures the filtering properties of the cotton.

In the report presented to the Academie des Sciences by M. Gorselin, in behalf of the committee above mentioned, although the theory of M. Guerin is left as an open question, the practical value of the dressing is abundantly recognized, and recommended to the careful attention of surgeons. The general opinion is that the cotton-wool is particularly useful as a dressing in amputations of the limbs when not too high up, less desirable in compound fractures and resections, and to be rejected in amputations of the breast.

The Boston Medical and Surgical Journal, August 27, 1874, contains a communication* from Thos. B. Curtis, M.D., of Boston, entitled "Cotton-Wool Dressing for Wounds." The practical details of the treatment, and other facts connected therewith, are thus set forth in Dr. Curtis' paper:

In December, 1870, Alphonse Guerin, Surgeon of the Paris Hospitals, now at the head of the Surgical Service at the Hotel Dieu, instituted a new dressing for wounds, consisting, mainly, in the use of cotton-wool, which is applied in very large quantities around the wounded limb, rendered firm and compact by tightly applied bandages, and left undisturbed for *several weeks.*

During the Franco-Prussian War, and during the Commune, the mortality among the wounded soldiers in Paris was very great, chiefly from pyæmia and other forms of hospital infection. Dr. Guerin was led to make experiments, with a view to discover some means of averting the terrible complications which make death

* Read before the Massachusetts Medical Society, June 2, 1874.

the almost inevitable result of every gun-shot wound, however treated. Believing in the germ theory of putrefaction and of septicæmia, and knowing the power of cotton-wool to deprive air of the putrefactive particles which it carries, he devised the cotton-wool dressing, and at once put it in practice in hospitals where his own practice and that of his colleagues had, till then, given the most disastrous results. His efforts were crowned by immediate success; and other surgeons, surprised at seeing his cases of amputation on the way to recovery, soon followed his example, and were similarly rewarded. Tyndall had already shown, by his experiments with a beam of sunlight traversing a dark space, that a sufficient thickness of cotton wool would arrest the solid particles conveyed by air in the shape of dust; and Pasteur had established the fact that urine, boiled and put away in flasks stopped up with cotton-wool, did not undergo putrefaction.

For surgical dressings, the wool itself must be of good quality, tolerably white and clean, and free from foreign matter; if possessed of a glazed surface, this must be stripped off, and the sheets of wool, torn into long strips about a foot wide, must be rolled up so that they may be methodically applied around the part to be dressed. It is difficult to believe what an enormous quantity of wool may be used in a single dressing. Guerin has sometimes used as much as four pounds. The usual fault is to apply too small a quantity of wool. Besides the wool in sufficient quantity, a number of rolled bandages, two inches wide and eight or ten yards long, must be at hand.

The supply of bandages must be ample, as may be recognized when it is known that the bandages removed from some of Guerin's dressings have amounted in all to 150 or 200 yards.

The operation and the dressing should never be performed in a septic atmosphere; never in the wards. The wool should be kept in bales, out of reach of contamination by the air of the surgical ward.

Guerin expressed his intention of following the advice of Pasteur, to purify his wool by subjecting it to a temperature of about 400° F. After cessation of all hæmorrhage, the wound is washed out with a one per cent. solution of carbolic acid. Guerin uses silk ligatures and cuts the threads off short, except one end of the ligature which holds the main artery.

The first step varies according as immediate union is or is not to be sought. In the first case, sutures can be used, unless the surgeon has acquired sufficient skill in the application of the dressing to be able to secure accurate coaptation of the flaps by the pressure of the cotton. If immediate union is not to be attempted, and such is the practice generally preferred by Guerin, the space between the flaps is to be completely filled with little wads of loose cotton. Then the sheets and rollers of cotton-wool are to be wrapped and wound over and around the limb, evenly and methodically, so as to surround it with a homogeneous mass of even thickness, which must in all cases extend beyond the first joint above the seat of amputation.

To fix the cotton-wool securely by means of the bandages so as to prevent its getting displaced, and to apply, through its superincumbent mass to the parts beneath, a very considerable degree of elastic compression, is in Guerin's eyes, a very important factor of success in the use of his dressing. Gradually the cotton-wool gets covered at every point by the successive overlapping turns of the roller, which grows tighter and tighter as the application progresses. When completed, the dressing forms a firm, smooth, hard mass, of uniform consistency.

If all go well, the dressing may be left undisturbed for two or three weeks. The *state of the dressing*, and *the general condition of the patient*, says Dr. Curtis, will furnish the requisite information as to the condition of the wound.

Guerin formerly reapplied the wool, sometimes two or three times, till the wound was completely healed, but it has been recognized that *after a certain stage of reparation was reached, further progress was very slow under the cotton-wool.* His practice now is to remove the first dressing at the end of the second, third or fourth week, then if the bone be well covered, the space between the flaps filled to a great extent with granulations, and the sanitary condition of the wards tolerably good, he brings the flaps together with plaster, and applies an ordinary dressing.

Dr. Curtis concludes his interesting paper with the following brief recapitulation of the merits and demerits of Guerin's dressing:

*Advantages.*—1st. Entire or partial prevention of pyæmia, erysipelas, and hospital gangrene. 2nd. Painlessness. 3rd. Immobility of the injured parts, to which, with the firm but even compression, is due the antiphlogistic action of the dressing. 4th. The rarity of the dressings. 5th. The transportability of the patient, whose injured limb is protected from all chances of mechanical injury.

*Disadvantages.*—1st. The necessity of ceaseless watching of every case by all the means at our command, including the daily use of the thermometer. 2nd. The slowness with which the last stages of reparation are effected under the cotton-wool. 3rd. The more or less disagreeable smell which the dressing generally acquires.

The cotton, it is said, acquires the disagreeable smell when applied to a sound limb for the same length of time.

